# Development of low glycemic index instant *Phirni* (pudding) mix-its visco-thermal, morphological and rheological characterization

**DOI:** 10.1038/s41598-022-15060-6

**Published:** 2022-06-23

**Authors:** Bazila Naseer, Haroon Rashid Naik, Syed Zameer Hussain, Tahiya Qadri, Basharat Nabi Dar, Tawheed Amin, Monica Reshi, Fouzia Shafi, Tabasum Fatima

**Affiliations:** 1 Division of Food Science and Technology, Sher-e-Kashmir University of Agricultural Sciences and Technology of Kashmir, Shalimar, 190025 India; 2 Department of Food Technology, Islamic University of Science and Technology (IUST), Awantipora, 192122 India; 3 Division of Basic Science and Humanities, Sher-e-Kashmir University of Agricultural Sciences and Technology of Kashmir, Shalimar, 190025 India; 4 Department of Moalijat, Regional Research Institute of Unani Medicine, Nasim Bagh, Srinagar, J&K 190006 India

**Keywords:** Nutrition, Health care

## Abstract

High amylose rice (HAR) and carboxymethyl cellulose (CMC) are the preferred choices for enhancement of resistant starch content and lowering of glycemic index in dairy desserts. The effects of different levels of skimmed milk powder (SMP): HAR flour (45:55 to 75:25) and CMC (0.1 to 1%) were investigated on physical characteristics of dry-mix and on texture profile parameters, resistant starch (RS), predicted glycemic index (pGI), glycemic load (GL) and overall acceptability of *phirni* (a traditional milk pudding). Design expert predicted SMP (70): HAR (30) and CMC (0.8%) as optimum levels for reducing the pGI and maximizing the RS content and other quality characteristics in *phirni.* RS content of *phirni* (4.38%) prepared from optimized dry-mix (ODM) was higher while pGI (48.12) and GL (7.50) were lower as compared to *phirni* prepared from market dry-mix (MDM). The visco-thermal properties of ODM and MDM also showed significant variations. Storage modulus (Gʹ) and loss modulus (Gʹʹ) indicated that ODM *phirni* was less solid than MDM *phirni*. Scanning electron micrographs showed fused structures in ODM, while coarse sheet like structures were observed across the surface of MDM. Thus, ODM can be a promising substitute for the available milk desserts for diabetic patients.

## Introduction

Traditional dairy based desserts have deep cultural preferences, but their manufacturing has been restricted to non-industrial sector only. *Phirni—*a classic creamy sweet pudding*,* is one of the most famous traditional dairy based dessert relished in Northern India, especially on festive and social occasions. *Phirni* is usually prepared from rice flour or semolina and milk with the addition of sugar, and flavoring agents. It is cooked to semi-solid or pasty consistency, by heat desiccation and consumed after brief refrigeration or cooling^1^. *Kheer* is used as a synonym for *phirni* in literature but the principal difference between the two is use of pregelatinized rice grains in former and rice flour in latter^2^. Dairy based traditional desserts have a very short shelf life without refrigeration, which has prompted researchers to develop technologies for making milk-cereal based pre-mixes with instant reconstitution. Jha et al.^2^ prepared ready-to reconstitute *Kheer* pre-mix from milk-rice slurry through spray drying. Different variants of Kheer mix have been prepared by substituting milk with skimmed milk (SMP)/whole milk powders (WMP)/soymilk and rice with other cereals besides addition of various functional ingredients^3,4^. Kumar et al.^5^ formulated *phirni* mix from pregelatinzed rice flour, sugar, cardamom powder and WMP:SMP in the ratio of 18, 31, 1 and 50:50%. The authors standardized cooking time of 10 min for preparation of *phirni*on the basis of sensory score and rheological parameters.

The global demand for low GI foods has increased many-folds due to increased prevalence of diabetes mellitus. The prevalence of diabetes mellitusis high with about 463 M people suffering from diabetes worldwide^6^. Rice serves as the main daily caloric source for more than half of the population worldwide, while its high increases the risk of diabetes mellitusdue to its high GI^7^. Although various low GI foods have been developed, but the efforts to develop low GI desserts are rare^8^.*Phirni* is a rich source of proteins and minerals. Also being semi-solid in nature, it is highly recommended for patients with swallowing difficulties. Rice is categorized as high GI food with GI score ranging from 40 to 100. However, rice starch having high amylose content (> 25%) usually tends to elicit a lower GI^9^. Gelling polysaccharides and gums are usually added to milk-based puddings for improvement of body and structure^10,11^. Zahidi et al.^10^ studied the effects of soy milk (10–100%) and CMC (0, 0.5%) on color and rheological properties of soy dessert. Samples containing CMC showed high consistency coefficient. Arancibia et al.^11^ studied the effect of CMC at concentrations ≤ 0.9 w/w on rheology and microstructure of soy protein desserts and concluded that texture and rheological characteristics of high protein desserts could be altered by adding thickeners. Carboxymethylcellulose (CMC)—a linear chain polymer of D-glucopyranose and anionic carboxylate groups is one such gelling polysaccharide. It has a unique property to limit availability of water which reduces gelatinization of starch^12^. The gelatinization behaviour of starch is directly influenced by the chemical and morphological structure of the gum present in the starch gel matrix, the swelling power of the granules, and the electrostatic interactions between starch granules and hydrocolloid molecules^13^. The interaction between starch and hydrocolloids can alter food texture, structure, and viscosity, thereby changing the accessibility of enzymes to starch granules^14^. Hydrocolloids have the potential effect to increase the viscosity of food products and alter the accessibility of starch granules to the α-amylase. Blending starch with hydrocolloids decreases the rate of starch digestion. Some previous studies have also reported that hydrocolloids decreased starch hydrolysis in rice starch^15^; corn starch^16^; maize and wheat starch mixtures^17^. Srikaeo and Paphonyanyong^18^ reported that addition of 1% hydrocolloids exhibited lower starch digestion rate in cooked rice samples. Oh et al.^19^ also reported that addition of 0.4% CMC altered the starch digestibility of dry heat treated high amylose rice.

Despite the increasing demand for low GI foods, no study has been conducted so far on starch digestibility and glycemic response of traditional dairy based dessert like *phirni*. Thus, the aim of this study was to a) investigate the effect of different levels of high amylose rice (HAR), skimmed milk powder (SMP) and carboxymethyl cellulose (CMC) on physical attributes of instant *phirni* dry-mix as well as on the textural parameters, resistant starch, glycemic index, glycemic load and overall acceptability of *phirni* reconstituted thereof, and b) compare the pasting, thermal, rheological and morphological properties of optimized dry-mix (ODM) and market dry-mix (MDM)*.* The hypothesis postulated in the study was that low GI traditional rice pudding “*phirni*” with improved quality characteristics can be prepared with ingredient alterations.

## Material and methods

### Raw material

Broken white grains of HAR (var *Lalat*) were ground in a laboratory mill (Perten, USA) to obtain rice flour that passed through 60 mesh sieve size. Moisture, protein, fat, crude fiber, dietary fiber, ash content and amylose content of rice flour were recorded as 11.30%, 10.15%, 1.25%, 1.09%, 3.90% and 0.56%, and 28.31%, respectively. The raw material was procured from the registered seed centers, and all the methods used in this work are in compliance with the institutional guidelines. Food grade CMC was procured from Sigma Aldrich, while SMP (Sifti, India), and market dry mix (MDM)were procured from the departmental store in Srinagar, India. In MDM, the ingredients were rice flour, milk powder, sugar, almonds and preservatives.

### Experimental design

The 5-level-2-factor central composite rotatable design (CCRD) was used to investigate the effects of different levels of SMP: HAR and CMC on physical characteristics of instant *phirni* dry mix, as well as on texture profile parameters, resistant starch (RS), predicted glycemic index (pGI), glycemic load (GL) and overall acceptability of *phirni* re-constituted from dry-mix. The experimental ranges of independent variables and their coded levels are illustrated in Table 1. Least square regression method was used to analyze the data and second order polynomial models were established using statistical software Design-Expert 9 (Stat-Ease Inc, Minnneapolis, MN, USA).

1 $$ y_{i} = b_{o} + \mathop \sum \limits_{i = 1}^{2} b_{i} x_{i} + \mathop \sum \limits_{i = 1}^{2} b_{ii} x_{i}^{2} + \mathop \sum \limits_{i = 1}^{2} \mathop \sum \limits_{i = 1}^{2} b_{ij} x_{i} x_{j} $$

where $y_{i}$ = response variable, x_i_ (i = 1, and 2), x_i_^2^ and x_i_x_j_ are linear, quadratic and interaction effects of the independent variables and b_o_, b_i_, b_ii_ and b_ij_ are regression coefficients for intercept, linear, quadratic and interactive effects, respectively. From ANOVA, the F-values were used to test the adequacy of models, while the model terms having “Prob > F” less than 0.05 were considered as significant terms^20,21^.

**Table 1:** Experimental ranges and coded levels of the independent variables generated using central composite rotatable design.

Run no	SMP:HAR (%)	CMC (%)
1	50:50 (− 1)	0.25 (− 1)
2	70:30 (+ 1)	0.25 (− 1)
3	50:50 (− 1)	0.85 (+ 1)
4	70:30 (+ 1)	0.85 (+ 1)
5	45:55 (− 1.414)	0.5 (0)
6	75:25 (+ 1.414)	0.5 (0)
7	60:40 (0)	0.1 (− 1.414)
8	60:40 (0)	1 (+ 1.414)
9	60:40 (0)	0.5 (0)
10	60:40 (0)	0.5 (0)
11	60:40 (0)	0.5 (0)
12	60:40 (0)	0.5 (0)
13	60:40 (0)	0.5 (0)

Coded values are in parenthesis.

*SMP* skimmed milk powder, *HAR* high amylose rice flour, *CMC* carboxymethyl cellulose.

### Preparation of instant *phirni* dry mix

All the dry ingredients i.e., HAR flour, SMP and CMC were mixed and homogenized in a planetary mixer (Phillips, India) in various proportions as per the experimental design (Table 1). Sucralose was added @ 680 mg/100 g dry mix weight in all the formulations^20,21^.

### Reconstitution of instant *phirni* dry-mix

The contents of *phirni* dry mix samples were mixed with potable boiled water in the ratio of 1:5 (*phirni* dry mix: water)^4^ in an open pan and subjected to cooking at low heat (80–85 °C) with constant stirring for 15-20 min till desired consistency was achieved. Reconstituted *phirni* was poured into plastic cups and kept at 4 ± 2 °C under refrigerated conditions for 12 h prior to analysis.

### Determination of physical attributes of instant *phirni* dry-mix

#### Bulk density, true density and solubility

Bulk density ($\rho_{B}$) and true density ($\rho_{T}$) were calculated by the method described by Raigar and Mishra^22^. Porosity was calculated using the standard formula^2^. The solubility percentage was calculated as per the procedure reported by Seth et al.^23^.

### Determination of textural, chemical and sensory properties of reconstituted *phirni* samples

#### Texture profile analysis

Texture profile analysis of the *phirni* samples was done using the texture analyzer, TA-XT2i (Stable Microsystems, Surrey, UK). The two-cycle penetration test at a penetration speed of 2 mm s^−1^, up to a depth of 5 mm was carried out with a 0.05 N load cell and P-20 stainless steel cylindrical probe^24^. The parameters recorded were hardness, cohesiveness (COH) and adhesiveness (ADH).

#### Resistant starch

The reconstituted *phirni* samples were freeze dried in a laboratory freeze drier (Leybold-Heraeus, GT 2A, Germany) and subjected to fine grinding. Grinded samples (100 mg) were incubated in a shaking water bath with pepsin (Roche, Germany), pancreatic alpha-amylase (Sigma-Aldrich, UK) and amyloglucosidase (Megazyme, 3,300U/mL) for 16 h at 37 °C as per the detailed protocol reported by Naseer et al.^21^. Resistant starch content was measured using Megazyme Assay Kit (Megazyme International, Wicklow, Ireland)^25^ and RS was calculated from the formula given in the instruction manual.

#### Predicted glycemic index and glycemic load

The procedure reported by Naseer et al.^21^ was followed to determine the in-vitro digestibility of different freeze dried *phirni* samples. The rate of starch digestion was expressed as the percentage of total starch (TS) hydrolyzed at different time intervals. Hydrolysis index (HI) was calculated using the starch digestion rate curve (glucose release). The area under the curve for the experimental sample divided by the area under the curve of the control sample (white bread) was taken as HI and predicted glycemic index (pGI) was calculated using the standard formula^26^

2 $$ {\text{pGI}} = {39}.{71} + \left( {0.{549} \times {\text{HI}}} \right) $$

Glycemic load (GL) of the samples was calculated using the following equation

3 $$ {\text{GL}} = { }\frac{{{\text{GI}} \times {\text{available }}\;{\text{CHO}}\;{\text{per }}\;{\text{serving}}\;{\text{ size}}}}{100} $$

where available carbohydrate (CHO)/serving size of 50 g was calculated by subtracting the dietary fiber content from total carbohydrate content.

#### Overall acceptability

Sensory evaluation of different reconstituted *phirni* samples was carried out on 9-point hedonic scale (9—liked extremely and 1—disliked extremely)^27^ by a jury of 30 trained judges. Samples were randomly coded and presented to the judges in separate partitioned booths. The judges evaluated the samples for different sensory attributes (appearance, consistency, flavor and lumpiness) according to the rating criteria and overall acceptability (OA) was determined as average of different sensory attributes. The judges used potable water for palate cleaning before evaluating each sample.

### Process optimization and validation

Specific goals were allocated to all the analyzed parameters to optimize the process for preparation of instant *phirni* dry-mix using desirability function approach. Among the physical and textural attributes, BD, TD, hardness, and cohesiveness were minimized; whereas, porosity, solubility, adhesiveness, and OA were maximized. RS content was also maximized, while pGI and GL were minimized. Out of different generated solutions, the solution with highest desirability value was selected for the preparation of instant *phirni* dry-mix. The actual values of physico-chemical, textural and sensory properties determined after evaluating the instant *phirni* dry-mix prepared from optimized ingredient levels were compared with the predicted values generated by the software to validate the optimization process. Percentage prediction error was calculated using the equation given by Scheuer et al.^28^.

### Quality evaluation of optimized dry-mix (ODM) and market dry-mix (MDM)

#### Physico-chemical analysis

Moisture, protein, ash, crude fiber, dietary fiber and fat contents were determined according to the standard methods of AOAC^29^. Water activity was measured with the help of water activity meter (Pre‐Aqua Lab, India). Carbohydrate content was estimated by difference method, and caloric value was calculated using Atwater factors. Total solids were estimated by gravimetric and total sugars were estimated using Lane- Eynon method as described by FSSAI^30^. Standard AACC^25^ method was followed to measure the total starch content using the total starch assay kit (K-TSTA, Megazyme, Bray, Ireland). Standard protocol was followed to determine the amylose content using the Megazyme assay kit (K-AMYL 06/18).

#### Visco-thermal properties

Pasting properties of different samples (3.50 g dry mix, 25 mL deionized water) were measured using rapid visco analyzer (RVA Starch TM, New Port, Scientific Warrie Wood, Australia) as per the procedure described by Pracham and Thaiudom^24^. The thermal properties were studied with a Differential Scanning Calorimeter (DSC-1 STARe 167 System, Mettler-Toledo). 10 mg of dry sample was placed in an aluminum pan and 50 µl deionized water was added. An empty aluminum pan was used as a reference. The pans were sealed hermetically and heated at 10 °C/min from 20 to 150 °C heating regime.

#### Rheological properties

Dynamic rheological properties were measured by Modular Compact Rheometer (MCR-101, Anton Paar, Austria), equipped with a parallel-plate geometry (50 mm diameter) using the procedure given by Thaiudomand Pracham^31^ with slight modification. The sample was placed on the ram of the rheometer with a spatula, spread uniformly andtrimmed wherever required. Storage modulus (G´), loss modulus (G´´), and loss tangent (tan δ) were analyzed by performing frequency sweep test from 0.1–100 rad/s, with a gap of 1 mm at 25 °C.The strain was kept constant at 2% for all the measurements which were in conformity with the Linear Viscoelastic Range.

#### Scanning electron microscopy

Morphology of the samples was analyzed by scanning electron microscope (Hitachi S-3400 N, Tokyo Japan) at 1.50 kX magnification. The samples were fixed on aluminum stubs using double sided adhesive tape. The fixed samples were covered with a thin layer of gold–palladium sputter coating and examined under voltage of 20 kV.

### Statistical analysis

Experiments were conducted in triplicate and results presented were average of three replications ± standard deviation. Statistical significance of physico-chemical, textural, pasting, and thermal properties was determined by Students t-test using SPSS software. Mean values were compared by Duncan’s Multiple Range test at *p* < 0.05 level of significance.

## Results and discussions

### Fit summary for ANOVA

For bulk density, true density, hardness, cohesiveness, resistant starch, predicted glycemic index, quadratic models were suggested; while for porosity, adhesiveness, glycemic load and overall acceptability linear modelswere suggested by output of fit summary statistics (Table 2). R^2^ values (0.834–0.990) recorded for different parameters indicated fair fit of developed models with the actual values. Models generated for different quality attributes of instant *phirni* dry mix were highly significant (*p* ≤ 0.0001). The difference in predicted and adjusted R^2^ was less than 0.2 in all the models which indicated that they are in reasonable agreement with each other^32^. F-values obtained (25.29 to 144.12) further demonstrated the validity of the models and there could be 0.01% possibility only that these F-values could be noise based. Range of coefficient of variation (CV = 0.22–10.97%) also confirmed the reproducibility of the developed models. The adequate precision values recorded for different parameters were considered desirable^33^. Lack of fit (LOF) was non-significant in all the selected parameters, indicating good correlation between second-order polynomial models and the measured data.

**Table 2:** Analysis of variance (ANOVA) for the developed regression models.

Coefficient	BD	TD	Porosity	Solubility	Hardness	ADH	COH	RS	pGI	GL	OA
Intercept	663.37	781.97	56.28	94.75	0.372	0.480	0.528	2.57	50.86	8.73	8.09
A-Skimmed milk powder	− 22.83**	− 33.09**	0.368**	1.95**	− 0.173**	0.075**	− 0.054**	− 0.052^NS^	− 0.088^NS^	− 1.17**	0.357**
B- Carboxymethyl cellulose	− 0.166^NS^	1.41^NS^	0.060^NS^	1.47**	− 0.061*	0.073**	0.074**	1.06**	− 1.45**	− 0.315**	0.262**
AB	− 2.03NS	− 0.985^NS^	–	− 0.270^NS^	0.014^NS^	−	0.023^NS^	0.169*	− 0.879**	–	–
A^2^	− 8.58**	− 9.14**	–	− 3.65**	0.042^NS^	–	0.007^NS^	0.589**	− 0.712**	–	–
B^2^	1.48^NS^	0.778^NS^	–	− 0.650^NS^	0.006^NS^	–	0.120**	0.236**	− 0.619**	–	–
Model *p*-value	< 0.0001	< 0.0001	< 0.0001	< 0.0001	< 0.0001	< 0.0001	< 0.0001	< 0.0001	< 0.0001	< 0.0001	< 0.0001
R^2^	0.963	0.984	0.881	0.969	0.952	0.872	0.959	0.990	0.961	0.936	0.834
Predicted R^2^	0.768	0.907	0.808	0.813	0.730	0.751	0.844	0.943	0.923	0.861	0.664
Adjusted R^2^	0.937	0.973	0.857	0.947	0.919	0.846	0.930	0.983	0.933	0.923	0.801
C.V %	0.80	0.61	0.22	0.930	10.97	7.91	5.85	4.48	0.81	3.33	2.26
F-Value	36.88	88.33	37.08	44.06	28.32	34.07	33.00	144.12	34.52	73.63	25.29
Adequate Precision	19.81	30.51	18.19	21.04	15.77	16.62	19.10	34.56	16.80	25.09	14.17
LOF	NS	NS	NS	NS	NS	NS	NS	NS	NS	NS	NS
Model analyzed	Quadratic	Quadratic	Linear	Quadratic	Quadratic	Linear	Quadratic	Quadratic	Quadratic	Linear	Linear

**Significant at *p* < 0.01, * Significant at *p* < 0.05.

*NS* Non-Significant, *LOF* lack of fit, *CV* coefficient of variation, *LOF* lack of fit, *BD* bulk density, *TD* true density, *ADH* adhesiveness, *COH* cohesiveness, *RS* resistant starch, *pGI* predicted glycemic index, *GL* glycemic load, *OA* overall acceptability.

### Physical properties of instant *phirni* mix

#### Bulk density, true density and porosity

**Figure 1:**
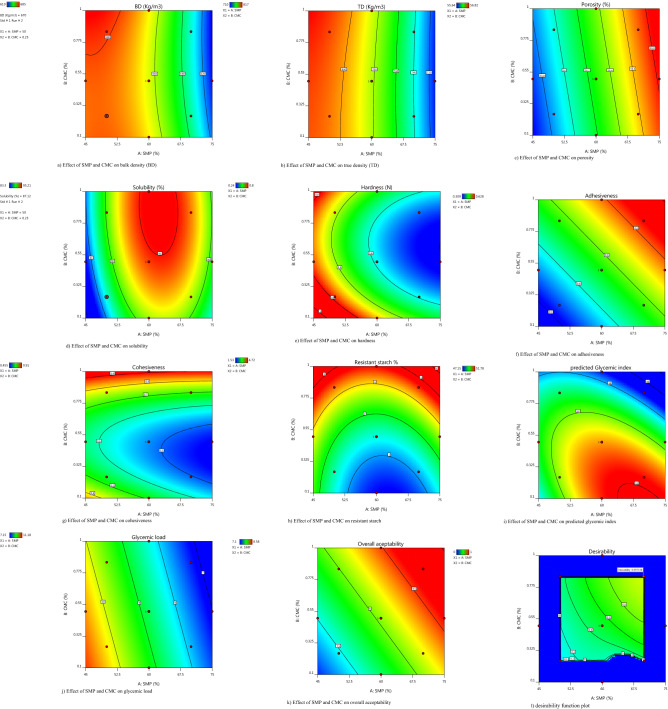
(**a**–**d**) Demonstrates the effect of skimmed milk powder (SMP): high amylose rice (HAR) and CMC on physical attributes of instant dry mix; (**e**–**k**) demonstrates the effect of SMP: HAR and CMC on textural parameters, resistant starch, kinetics of starch digestion and overall acceptability of instant dry mix *phirni,* and (**l**) optimization plot.

Bulk density (BD), true density (TD) and porosity ($\emptyset )$ are important functional parameters of instant powders from commercial point of view^34^. For different experimental runs, BD, TD and $\emptyset$ of *phirni* mix ranged from 610 to 685 kg/m^3^, 710 to 817 kg/m^3^ and55.64 to 56.82% respectively (Fig. 1a–c). Fitted regression models for BD, TD and $\emptyset$ are depicted below, where, A indicates proportion of SMP in relation to HAR flour

4 $$ {\text{BD}} = {663}.{37}{-}{22}.{\text{83A}}{-}{8}.{\text{58A}}^{{2}} $$

5 $$ {\text{TD}} = {781}.{97}{-}{33}.0{\text{9A}}{-}{9}.{\text{14A}}^{{2}} $$

6 $$ {\text{Porosity }}(\upphi ) = {56}.{28} + 0.{\text{368A}} $$

Fitted regression Eqs. (4–6) shown above indicated that SMP incorporation (A) had significant negative linear and quadratic effects on bulk density and true density of *phirni* mix. However, linear effects of SMP were predominant over quadratic effects in both BD and TD. SMP had significant (*p* < 0.05) linear effect on porosity of *phirni* mix as well. As the concentration of SMP was increased, BD and TD were decreased, while ϕ increased (Fig. 1a–c). SMP has lower density than rice flour, which might have reduced the BD of *phirni* mix samples containing higher proportion of SMP. Thus, agglomeration of particles was more in the *phirni* mix samples containing higher proportion of SMP which justified the inverse and direct relationships of SMP with density and porosity, respectively. The density values of *phirni* mix recorded in the present study were lower, while porosity percentage was higher than that reported by Jha et al.^2^ for kheer mix, likely due to compositional and ingredient variation.

#### Solubility

Solubility is also one of the important quality attributes of instant powders. Solubility of different *phirni* mix samples varied from 83.3 to 95.2% (Fig. 1d). Fitted regression model for solubility is depicted below, where, A indicates proportion of SMP in relation to HAR flour and B is concentration of CMC

7 $$ {\text{Solubility}} = {94}.{75} + {1}.{\text{95A}} + {1}.{\text{47B}}{-}{3}.{\text{65A}}^{2} $$

Regression Eq. (7) exhibited positive linear relationships of A and B and a negative quadratic relationship of A with solubility. As the concentration of SMP was increased from 45 to 60%, solubility increased from 83.3 to 95.16%. However, upon further increase in SMP concentration (60 to 75%), the solubility of *phirni* mix decreased from 95.16 to 88.18%, respectively (Fig. 1d). Solubilization of instant dairy mix is governed by structural organization of casein proteins and its interactions with water^35^. SMP has higher dispersibility and porosity and thus, produces lower sediment value as compared to rice flour, which may have increased the solubility of *phirni* mix developed from blends containing 45–60% SMP. However, higher incorporation of SMP increases the protein content of the blends, which might have decreased the solubility of *phirni* mix containing greater than 60% SMP, due to hydrophobic protein–protein interactions^36,37^ and protein-hydroxyl interactions of SMP and CMC^38^. McSweeney et al.^36^ also reported inverse effect of protein content on solubility of milk concentrates. Moreover, BD and solubility have inverse relationship^39^ and same trend was noticed in the present study as well (Eq. 4 and Eq. (7), respectively). Equation (7) and Fig. 1d also demonstrated the significant positive linear effect of CMC concentration on solubility of dry mix. As the concentration of CMC was increased from 0.1 to 1%, solubility increased from 90.31 to 95.21%, respectively (Fig. 1d). The presence of hydroxyl groups facilitates the bonding between CMC and neighboring water molecules, which may have increased the solubility of *phirni* mix samples containing higher levels of CMC. Although CMC is highly hygroscopic in nature, however, interaction of proteins and hydroxyl groups induce some conformational changes which may have led to difference in sensitivity of *phirni* mix to hydration and binding of water molecules^38^.

### Physico-chemical properties of reconstituted *phirni*

#### Texture profile analysis

##### Hardness

Hardness of different reconstituted *phirni* samples ranged from 0.24 to 0.80 N (Fig. 1e). Fitted regression model for H is depicted below:

8 $$ {\text{Hardness}} = 0.{372}{-}0.{\text{173A}} - 0.0{\text{61B}} $$

Regression Eq. (8) indicated that A had a significant negative linear effect on hardness. As the concentration of SMP increased from 45 to 75%, hardness decreased from 0.80 to 0.24 N, respectively (Fig. 1e). Incorporation of SMP lowered the starch content of the formulation which may have decreased the hardness of *phirni* prepared from such samples. At the highest concentration of rice flour (55%), maximum hardness was recorded highest (0.80 N), which could be attributed to interaction of SMP proteins with leached components of starch in *phirni* during cooling^40^. Amylose has an ability to form rigid gel, which may have caused interaction starch and milk causing positive affect on the firmness of puddings^41^. Figure 1e shows that B also had significant negative linear effect on hardness. As the concentration of CMC was increased from 0.1 to 0.5%, hardness decreased from 0.75 to 0.36 N. CMC has good holding capacity and thus decreases the gel hardness during cooking which subsequently leads to the production of soft puddings^42^.

##### Adhesiveness and cohesiveness

Adhesiveness (ADH) indicates the ability of the product to adhere to the palate during swallowing^43^ while cohesiveness (COH) measures the strength of internal bonds and the degree to which a food can be deformed before it breakdowns^44^. Both ADH and COH are important factors which govern the acceptability of milk desserts. ADH of different *phirni* samples ranged between (−) 0.309 to (−) 0.628 N and COH from 0.455 to 0.910, respectively (Fig. 1f,g). Fitted regression models for ADH and COH are depicted below:

9 $$ {\text{ADH}} = 0.{48}0 + 0.0{\text{75A}} + 0.0{\text{73B}} $$

10 $$ {\text{COH}} = 0.{528}{-}0.0{\text{54A}} + 0.0{\text{74B}} + 0.{12}0{\text{B}}^{{2}} $$

Both the independent variables (A and B) exhibited positive linear relationships with ADH (Eq. 9). Figure 1f indicated that as the SMP and CMC concentrations increased from 45 to 75% and 0.1 to 1%, the ADH of *phirni* samples increased from (−) 0.341 to (−) 0.563 N and (−) 0.309 to (−) 0.628 N, respectively. Higher ADH implies softer texture, which is a desirable trait in milk desserts. Casein—a dominant protein in SMP forms protein gel upon gentle heating and stirring and thus imparts body and texture to *phirni*^45^. Higher SMP reduces the consistency of *phirni* due to formation of liquid bridges, which increases the ADH. Furthermore, lactose act as a plasticizer in the presence of water due to its hygroscopic nature and thus, depresses the glass transition of amorphous sugars^46^, which also justifies the increase in ADH upon increase in SMP level in *phirni.* Guimarães et al.^47^ also reported a significant increase in ADH of instant dessert with the increase in addition of milk powder in relation to rice flour. The positive relationship of CMC and ADH recorded in *phirni* samples can be attributed to CMC-calcium ion interaction. Coronato et al.^48^ reported that with increase in gum concentration, the adhesiveness of milk-gum suspension also increased.

Regression Eq. (10) indicated inverse relationship of A and direct relationship of B with COH. The perusal of the results depicted in Fig. 1g demonstrate that increase in concentration of SMP from 45 to 75% decreased the COH from 0.651 to 0.455, while increase in concentration of CMC from 0.1 to 1% increased the COH from 0.721 to 0.910, respectively. Decrease in COH with the increase in SMP level can be attributed to dilution of starch network and weakening of interparticle bonding^49^ caused by easy dissolution of SMP in water. In contrast, CMC imposed a thickening effect in *phirni* which may have lead to increase in COH^50^ due to starch-milk interaction. Zhang et al.^51^ also reported that addition of gums increased the consistency and cohesiveness of the low-fat fermented skimmed milk.

#### Resistant starch

Resistant starch (RS) is an important contributor of glycemic response offered by carbohydrate-rich food; hence, lot of research is going on to enhance the RS content in processed foods due to its health benefits^52^. RS content of different *phirni* samples ranged from 1.53 to 4.72% (Fig. 1h). Fitted regression model for RS is depicted below:

11 $$ {\text{RS}} = {2}.{57} + {1}.0{\text{6B}} + 0.{\text{169AB}} + 0.{\text{589A}}^{{2}} + 0.{\text{236B}}^{{2}} $$

Both the independent variables exhibited significant positive relationships with RS content (Eq. 11). Interactive effect of A and B on RS content was also found to be significantly positive. The data depicted in Fig. 1h indicated that as the increase of SMP increased from 60 to 75%, the RS content increased from 2.25 to 3.44%, while as upon increase in CMC concentration from 0.1 to 1%, RS content increased from 1.53 to 4.62%, in different *phirni* samples respectively. Milk proteins interact with the rice starch granules through adsorption and hydrophobic linkages^53^. Adsorption of protein aggregates fill the voids inside starch granules which restrict the diffusion of water and thus, limit the starch hydrolysis and digestion. Further, heating induces protein–protein aggregation, and formation of amylose–lipid complexes which may reduce the accessibility to hydrolysis^54^. CMC exhibited a dominant effect on RS content as compared to SMP due to its thickening effect. A prominent increase in RS content with the increase in CMC concentration is evident in Fig. 1h. CMC binds to the surface of proteins due to the electrostatic attraction between the positive charge on casein micelles and anionic groups on the CMC molecules due to which enzyme–substrate interactions during enzymatic hydrolysis gets reduced. In addition, electrostatic interaction also leads to protein-polysaccharide complex formation which are relatively resistant to digestion^55^. Elmstahl^56^ also reported an increase in RS content in semolina porridge due to starch-protein interactions.

#### Predicted glycemic index and glycemic load

Low-GI foods are recommended by the American Diabetic Association for the long-term management of diabetes mellitus^57^. pGI and GL of different *phirni* samples ranged from 47.15 to 51.78 and 7.15 to 11.18, respectively (Fig. 1i–j). Fitted regression models for pGI and GL are depicted below:

12 $$ {\text{pGI}} = {5}0.{86}{-}{1}.{\text{45B}}{-}0.{\text{879 AB}}{-}0.{\text{712A}}^{{2}} {-}0.{\text{619B}}^{{2}} $$

13 $$ {\text{GL}} = {8}.{73}{-}{1}.{\text{17A}}{-}0.{\text{315B}} $$

Both the independent variables exhibited significant negative relationships with pGI and GL (Eq. 12 and 13). In case of pGI, the interactive effect of A and B was also significantly negative. The data depicted in Fig. 1i–j indicated that pGI of *phirni* samples decreased from 51.55 to 49.64 as the SMP concentration increased from 60 to 75%; while as upon increase in CMC concentration from 0.1 to 1%, pGI decreased from 51.78 to 47.18, respectively. At the same time, the increase in SMP concentration from 45 to 75% decreased the GL from 11.18 to 7.15 and increase in CMC concentration from 0.1 to 1%, decreased the GL from 8.96 to 8.22 in different *phirni* samples (Fig. 1i–j). Milk proteins located in the interfaces of starch granules limit the water diffusion inside the granules which restrict the gelatinization of the starch^40^. Proteins and partially/ungelatinized starch granules are less susceptible to hydrolysis and are thus involved in starch-protein interaction which lowers the glycemic response and glycemic load upon their co-ingestion^58^. Milk proteins are also known to have insulinotropic properties and thus, reduce the glycemic response^59^.Sugiyama et al.^60^ also reported that the consumption of rice and milk attenuates; Sun et al.^58^ also reported that co-ingestion of soymilk and dairy milk with bread significantly lowered the blood glucose levels. Out of two independent variables, CMC exhibited a dominant effect on pGI as compared to SMP (Eq. 12). Hydroxyl groups of CMC interact with starch granules—via—hydrogen bonding and form thermally stable structures. The decrease in pGI and GL could also be accorded to the enhanced structural stability of starch induced due to immobilization of water and crosslinking of CMC with glycosidic linkages during heating. Further, the hydrophilic polysaccharides due to its linear structure and ionic charge assist in formation of retrograded starch^61^ during cooling of *phirni.* Jung et al.^62^ also reported that glycemic index of segoami rice gels decreased significantly as the concentration of gums was increased from 0.3 to 0.7%.

#### Overall acceptability

The SMP and CMC formulations significantly affected the sensory attributes of *phirni*samples. For different *phirni* samples, overall acceptability (OA) ranged from 7.10 to 8.58 (Fig. 1k). Fitted regression model for OA is given below:

14 $$ {\text{OA}} = {8}.0{9} + 0.{\text{357A}} + 0.{\text{262B}} $$

The Eq. (14) depicts significant positive relationships of A and B with OA. Figure 1k also indicated an increasing trend in OA with the increase in concentration of SMP and CMC. SMP improved the flavor of *phirni*, possibly due to presence of milk sugar while CMC improved the sensory attributes like texture and viscosity, due to its thickening effect*.* Also, the starch gets gelatinized during cooking and interacts with milk proteins and hydrocolloid which may have further imparted the desired texture and mouthfeel to *phirni*^5^.

### Optimization

Design expert predicted high amylose rice flour (70): SMP (30) and CMC 0.85% as optimum ingredient levels for preparation of low GI *phirni*. The desirability value for the selected solution was 0.70 (Fig. 1l). The BD, TD, porosity, S, H, ADH, COH, RS, pGI, GL and OA of *phirni* prepared from optimized ingredient levels were recorded as 634.15 kg/m^3^, 745.10 kg/m^3^, 55.16%, 94.24%, 0.311 N, (−) 0.642 N, 0.724, 4.38%, 48.12, 7.50, 8.39, respectively. The actual values of dependent variables matched the predicted values well with a variation of ≤ 3.66% which confirmed the predicted pattern of the developed models.

### Chemical composition

Physico-chemical composition of optimized drymix (ODM) and market dry mix (MDM) is depicted in Table 3. Moisture, water activity, and total solid content of ODM and MDM differed non-significantly with each other. The values of these parameters were more or less similar to the values reported by Jha et al.^2^ and Vashistha et al.^63^ in different types of dairy dry-mixes. Protein content was significantly higher, while fat content was significantly lower in ODM as compared to MDM, possible due to ingredient difference. Higher ash content of ODM as compared MDM was presumable due to SMP. Crude and dietary fiber contents were also significantly higher in ODM than MDM, which might be attributed to incorporation of CMC in ODM. However, carbohydrate content was significantly lower in ODM as compared to MDM. Significant reduction in carbohydrate content of ODM was probably due to its higher protein, and fiber content, besides the lower total sugar content. In case of MDM, sucrose is usually added as a taste enhancer, while in case of ODM, lactose was the major sugar present due to SMP incorporation. Lactose has a GI score of 43 and falls under the low GI category, while sucrose has a GI score of 60 and falls under the moderate GI category^64^. Energy value of ODM was significantly lower than that of MDM, likely due to lesser sugar, fat and carbohydrate contents in the former. Significantly higher starch content of ODM than that of MDM might be because of high proportion of rice flour in ODM. Since high amylose rice flour was used as base material for preparation of ODM, which may have led to its high amylose content as compared to MDM. Variation in SMP, rice flour and other ingredients possibly led to observed disparities in compositional analysis of ODM andMDM. Higher RS content (4.38%) was recorded in *phirni* reconstituted from ODM as compared to MDM *phirni* (0.50%), which can be attributed to high amylose content and CMC incorporation in ODM. Due to linear chain structure, amylose is more susceptible to retrogradation during heating–cooling cycle, which might have increased the RS content in ODM *phirni*. In addition, various chemical changes take place during *phirni* preparation, which may have facilitated protein denaturation and formation of complexes between starch and non-starch components that are resistant to amylolytic hydrolysis^65^. The lower glycemic response of ODM *phirni* (48.12) relative to MDM *phirni* (60.20), was possibly due to its higher RS and dietary fiber content. The presence of sucrose may possibly have spiked the pGI of MDM *phirni* as well. Variation in glycemic index of *phirni* reconstituted from ODM relative to MDM highlighted the importance of rice and sugar type including the effect of hydrocolloid on postprandial hyperglycemic effect.

**Table 3:** Physico-chemical properties of optimized dry mix (ODM)and market dry mix (MDM).

Parameters	ODM	MDM
Moisture (g/100 g)	4.61^a^ ± 0.25	4.02^a^ ± 0.01
Water activity	0.26^a^ ± 0.21	0.31^a^ ± 0.10
Total solids (g/100 g)	95.20^a^ ± 1.05	95.90^a^ ± 1.55
Protein (g/100 g)	25.12^a^ ± 0.31	9.56^b^ ± 0.35
Fat (g/100 g)	1.30^a^ ± 0.25	2.14^b^ ± 0.15
Ash (g/100 g)	7.12^a^ ± 0.22	6.08^b^ ± 0.17
Crude fibre (g/100 g)	1.18^a^ ± 0.15	0.29^b^ ± 0.10
Total carbohydrate (g/100 g)	60.58^a^ ± 0.45	77.91^b^ ± 0.54
Total sugars (g/100 g)	30.00^a^ ± 1.88	50.25^b^ ± 2.50
Energy value (kcal/100 g)	354.50^a^ ± 1.9	369.14^b^ ± 1.0
Dietary fiber (g/100 g)	3.10^a^ ± 0.66	0.67^b^ ± 0.37
Amylose (g/100 g)	15.31^a^ ± 0.25	5.27^b^ ± 0.36
Total starch (g/100 g)	25^a^ ± 2.0	10^b^ ± 1.8
RS^1^ (g/100 g)	4.38^a^ ± 1.5	0.50^b^ ± 1.75
HI^1^	15.31^a^ ± 0.15	37.32^b^ ± 0.08
pGI^1^	48.12^a^ ± 1.1	60.20^b^ ± 1.50
GL^1^	7.50^a^ ± 0.11	9.78^b^ ± 0.16
Hardness^1^ (N)	0.311^a^ ± 1.8	0.654^b^ ± 1.5
Adhesiveness^1^ (N)	− 0.642^a^ ± 0.41	− 0.550^b^ ± 0.22
Cohesiveness^1^	0.724^a^ ± 0.66	0.789^b^ ± 0.82

^1^Parameters analyzed after reconstitutingODM and MDM.

Mean values in rows with different superscripts are significantly different at *p* ≤ 0.05.

*RS* resistant starch, *HI* hydrolysis index, *pGI* predicted glycemic index, *GL* glycemic load.

### Texture profile analysis

TPA of *phirni* reconstituted from ODM and MDM is shown in Table 3. Hardness and cohesiveness (COH) were higher in *phirni* reconstituted from MDM as compared to that reconstituted from ODM. Higher protein content reduces the leaching of amylose possibly leading to reduction of the hardness of ODM *phirni*. It can be presumed that network of casein micelles dominated the texture of ODM *phirni.* Equation (8) also indicated that SMP had dominant negative effect on hardness of *phirni* as compared to the positive effect of CMC. Further, due to presence of additional ingredients, MDM upon heating produced viscous pastes which converted into firm gel after cooling. In contrast, ODM during reconstitution produced liquid like consistency and thus, formed firm gel after cooling, which might have reduced the COH of ODM *phirni*. However, ODM *phirni* had higher ADH as compared to MDM *phirni*, possibly due to its softer texture and presence of lactose owing to high SMP (70%) incorporation^46^.

### Pasting properties

The pasting properties of ODM and MDM are depicted in Table 4. ODM showed significantly higher pasting temperature than MDM suggesting that starch crystallites formed in ODM were resistant to melting. Also, starch-hydrocolloid interaction might have created some stable structures, which require higher temperature to disorganize^66^. Noisuwan et al.^40^ reported that proteins adsorb onto the starch granules and restrict the diffusion of water into the starch granules during pasting, which may increase the pasting temperature of ODM. However, peak viscosity and hold viscosity were higher in MDM as compared to ODM (Table 4). Presence of sucrose in MDM may have contributed to its higher peak and hold viscosities, which is in agreement with the results of Pongsawatmanit et al.^67^ for sucrose incorporated starch suspensions. However, higher amylose content (15.31%) in ODM may have restricted the development of viscous paste and thus, significantly lowered its peak and hold viscosities. In addition, due to high protein content (25.12%), the protein molecules in ODM tend to encircle the starch granules in the matrix which hinders its water absorption. One of the possible mechanisms which can explain the influence of SMP on pasting behaviour of rice flour could be that proteins compete for water during gelatinization of starch and thus, restrict the swelling of granules. At the same time, lactose and salt diffuses into starch granules which affects the leaching of amylose into continuous phase by anti-plasticizing effect of lactose and stabilizing effect of cations on starch structure, respectively^24^. The paste has lower breakdown viscosity (BDV) and has better stability against shearing, which is a desirable trait for puddings^68^. The lower BDV of ODM indicated higher reinforcing effect of protein matrixes on starch-protein interaction, possibly due to disulfide bonding, which may have improved the shear resistance of ODM as compared to MDM. Final viscosity (FV) and setback viscosity (SBV) of ODM were significantly higher than that of MDM (Table 4). The higher FV of ODM can be attributed to the presence of CMC. The interaction between negatively charged CMC molecules and positively charged casein miscelles, controlled by electrostatic repulsion^69^ may have led to increase in FV. The results of pasting properties were almost in agreement with the results of Pracham and Thaiudom^24^ reported for jasmine rice pudding. Higher SBV recorded in case of ODM indicated its higher retrogradation tendency which was possibly due to its high amylose content. Ye et al.^70^ and Naseer et al.^71^ have also reported higher SBV for high amyloses rice.

**Table 4:** Pasting and thermal properties of optimized dry mix (ODM)and market dry mix (MDM).

	ODM	MDM
**Pasting properties**		
Pasting temperature (°C)	81.23^a^ ± 2.25	76.13^b^ ± 2.30
Peak viscosity (cP)	170^a^ ± 3.05	228^b^ ± 2.55
Hold viscosity (cP)	163^a^ ± 1.33	212^b^ ± 1.20
Breakdown viscosity (cP	7^a^ ± 0.88	16^b^ ± 1.66
Final viscosity (cP	321^a^ ± 3.75	272^b^ ± 2.15
Set back viscosity (cP	158^a^ ± 2.78	60^b^ ± 2.99
**Thermal properties**		
Onset temperature T_o_ (°C)	35.41^a^ ± 1.69	36.05^a^ ± 2.47
Peak temperatureT_p_ (°C)	77.63^a^ ± 1.55	73.80^b^ ± 1.79
Endset temperature T_c_ (°C)	126.82^a^ ± 3.12	97.72^b^ ± 2.66
Enthalpy of gelatinization (∆H_gel_) (j/g)	21.13^a^ ± 2.0	17.93^b^ ± 1.30

Mean values in rows with different superscripts are significantly different at *p* ≤ 0.05.

### Thermal behaviour

Differential Scanning Calorimetric (DSC) results presented in Table 4 showed that ODM had significantly higher peak, end-set temperatures and enthalpy of gelatinization (∆H_gel_) than MDM. However, onset temperatures recorded for both the samples showed non-significant difference. CMC, protein and amylose molecules compete with each other for water^72^, which may have enhanced the peak and end-set temperatures of ODM. Furthermore, high amylose starches also exhibit higher gelatinization temperatures^73,74^. Uthumporn et al.^72^ also reported that protein and starch granules compete for water molecules which results in inhibited swelling and increased gelatinization temperature. Higher ∆H_gel_ suggested that more organized amylose structures were present in ODM as compared to MDM and thus, higher energy was required to break the hydrogen bonds between the monomers in case of ODM.

### Microstructure analysis

**Figure 2:**
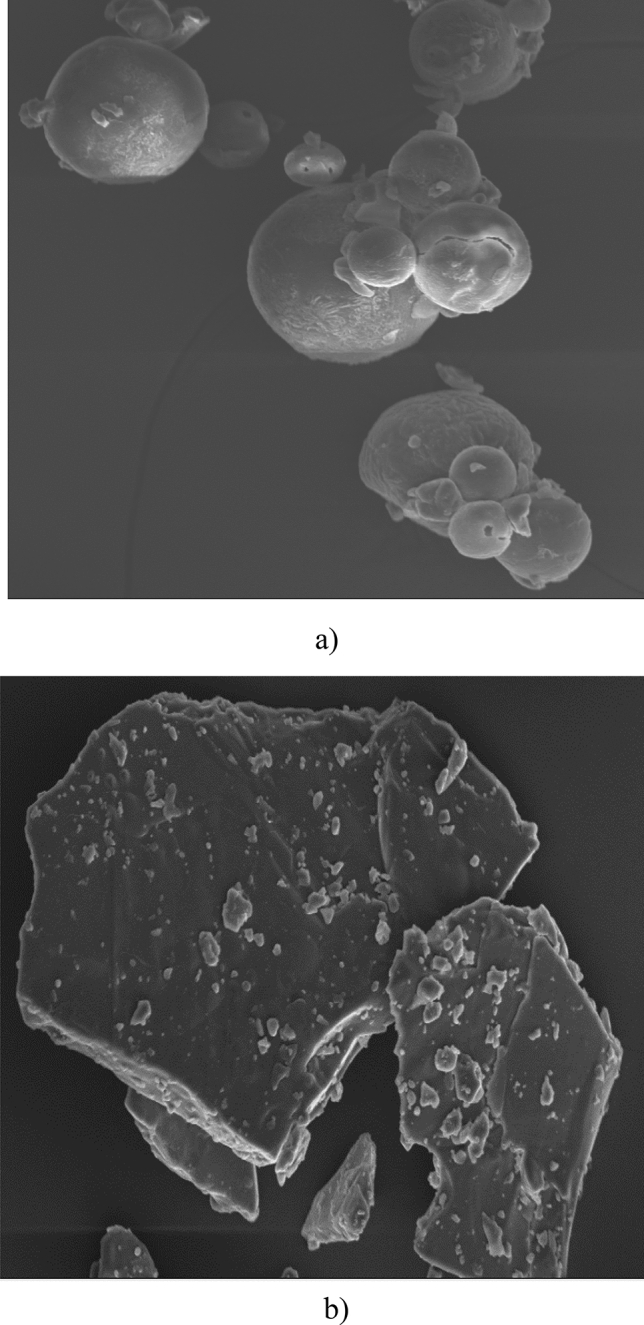
Scanning electron micrographs of (**a**) optimized dry mix (ODM), and (**b**) market dry mix (MDM).

Scanning electron micrographs given in Fig. 2a demonstrated the presence of fused structures in ODM. The starch granules possibly fused with protein molecules and formed aggregated complexes which appeared as fused structures. Further, the casein micelles linked to each other with inter-micellar bridges seemed to appear like balls in micrographs of ODM (Fig. 2a). Fine spikes around these casein aggregates can be attributed to the presence of polysaccharide/fibrous materials^75^. Overall, it appeared as if the surface of ODM was dominated by the proteins and different constituents of rice were encased by protein miscelles. Further, the interaction of CMC and casein particles may also have resulted in the agglomeration of particles, which may have acted as a physical barrier to enzymatic hydrolysis^76^. In contrast, coarse sheet like structures were observed in MDM (Fig. 2b). Some irregular shaped components and spherical protein bodies were found dispersed all over the surface of MDM, which could be attributed to difference of ingredients and processing conditions adopted for preparation of market sample. Borad et al.^77^ also reported coarse sheet like structures in different kheer samples.

### Rheological properties

**Figure 3:**
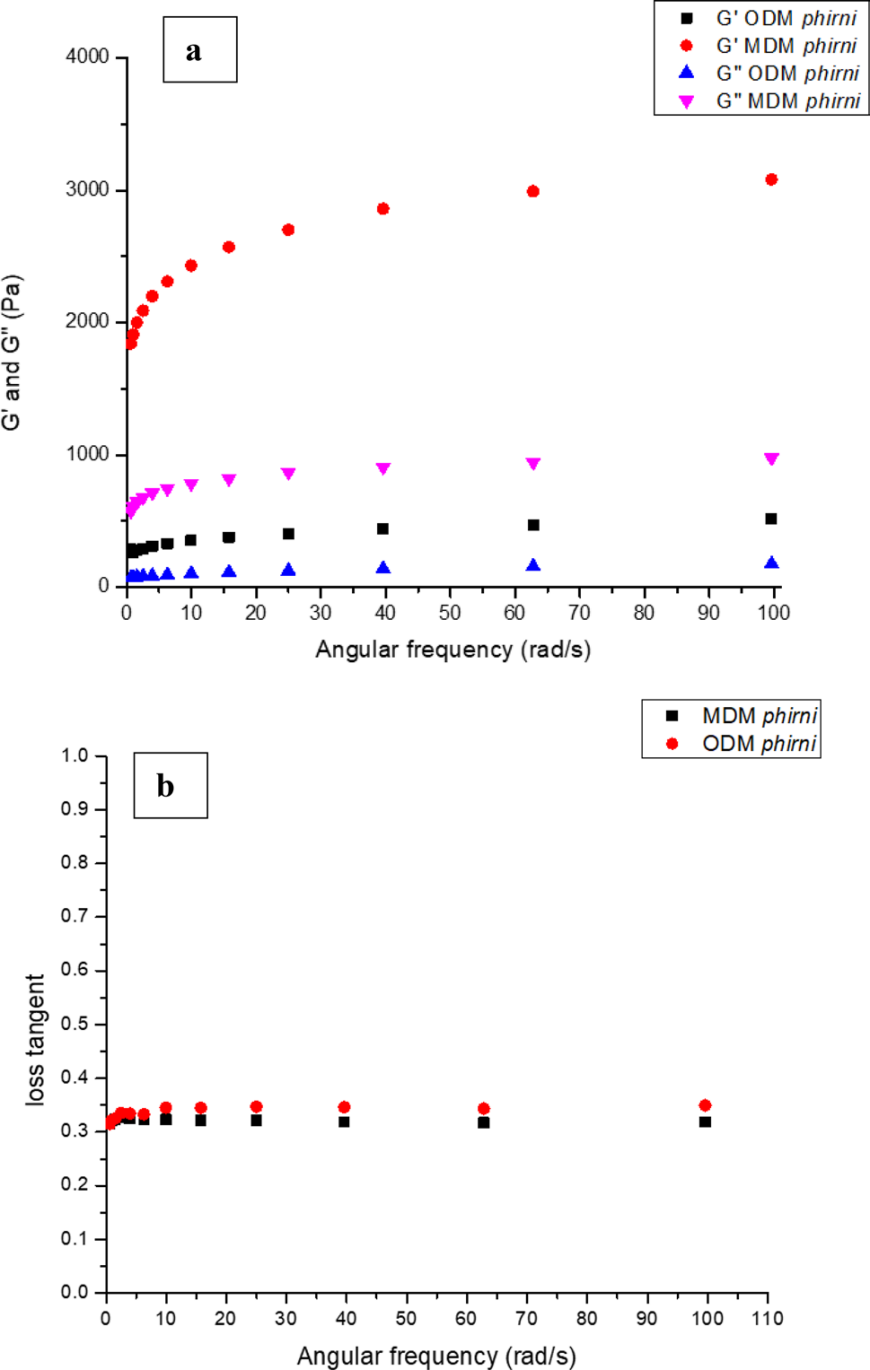
(**a**) Gʹ and Gʹʹ and (**b**) Loss tangent of *phirni* reconstituted from ODM and MDM.

Due to the presence of multiple components like proteins, sugar, additives and stabilizers, rice-milk desserts usually show somewhat complex rheological behaviour. Both ODM and MDM *Phirni* samples had G′ > G" and tan δ < 1 over entire range of studied frequency (Fig. 3a,b). These trends indicate a typical soft gel behaviour and have been observed with milk puddings^24,68^. Higher storage (G′) and loss modulii (G") were recorded for MDM *phirni* which indicated its more solid like behaviour as compared to ODM *phirni.* Due to flavoring agents and additives, the ingredient interaction was probably stronger in MDM *phirni* which might have increased its G′ and G". Therefore, it was presumed that total solids and other particulate matter exhibited the dominant affect on pudding structure and gel strength. Lower G′ and G" of ODM *phirni* indicated its less firm structure as compared to MDM *phirni* sample. Although, ODM was prepared from high amylose rice flour with CMC incorporation (0.85%), but the G′ and G" values indicated that ODM *phirni* was less solid-like as compared to MDM *phirni*. At low concentration (≤ 4%), hydrated CMC molecular chains act as “diluent” and disperse around amylose molecules, thereby, prevent the amylose-amylose interaction which may reduce the rigidity of the pastes^78^. Further, due to high SMP percentage, the casein micelles in ODM may also have restricted the interaction between amylose and other ingredients. It is likely possible that either amylose leaching may have been inhibited by casein aggregates or leached amylose granules would have been encased by casein network^40^. El-Garaway and Salam ^79^ also described that high protein concentration imposed hinderance on formation of strong gel network between amylose-amylopectin and CMC. Tan δ of ODM *phirni* was higher than MDM *phirni* (Fig. 3b), which also indicated that at high protein content, CMC indisposed the amylose network and reduced the rigidity of the paste. Milk proteins also prevent the molecular reorganization of high amylose starch and thus reduce the rigidity of gels as compared to low amylose ones. Carvalho et al.^80^ also suggested that casein micelles act as inactive fillers in starch granules and thus, produce weak gels. Since sucrose was present in MDM, that has tendency to create preferential exclusion of sugar and proteins which increases the effective concentration of sugars in bulk volume and therefore, increases the firmness and viscosity. However, in case of ODM, co-adsorption of rice flour components may have altered the surface charge of proteins. This may have reduced the protein–protein interaction and subsequently the gel strength of ODM *phirni*^81^. Pracham and Thaiddum^24^ also reported that high protein puddings exhibited weak gel structure as compared to low protein puddings. The rheological and texture profile results recorded for ODM and MDM *phirni* samples in the present study were in synchronization with each other. Similar trends were reported by Prachamand Thaiddum^24^ in milk based puddings.

## Conclusions

Perusal of the results revealed that addition of CMC can play a predominant role in enhancing the RS and lowering the pGI of traditional milk-based rice pudding-*phirni*. Physical attributes of optimized dry mix were dominantly affected by SMP, while RS and pGI were mainly controlled by CMC. However, both SMP and CMC seemed to work in synergy with high amylose rice in increasing the resistant starch and lowering the pGI of reconstituted *phirni*. Milk proteins played a vital role in pasting and thermal behaviour of optimized dry mix, which was validated by scanning electron microscopy as well. The elastic (G') and viscous (G") components confirmed that *phirni* reconstituted from both ODM and MDM exhibited soft gel behaviour. However, ODM *phirni* had less firm texture than MDM *phirni*. Texture profile analysis also indicated that ODM *phirni* had lower hardness but higher adhesiveness and cohesiveness as compared to MDM *phirni*. The outcome of the present study can help to prepare a low GI diet for people suffering from diabetes and swallowing difficult. However, this is the first reported study which investigated the glycemic index, glycemic load and resistant starch content of traditional dessert-*phirni.* Therefore, prior to its commercialization, there is a need to conduct in-vivo testing of such product among the target population in future to validate its impact on glycemia.

## Data Availability

The datasets analysed during the current study shall be made available from the corresponding author on reasonable request.
